# Enhancing LI-RADS Through Semi-Automated Quantification of HCC Lesions

**DOI:** 10.3390/jpm15090400

**Published:** 2025-08-29

**Authors:** Anna Jöbstl, Piera Maria Tierno, Anna-Katharina Gerstner, Gudrun Maria Feuchtner, Benedikt Schaefer, Herbert Tilg, Gerlig Widmann

**Affiliations:** 1 Department of Radiology, Medical University of Innsbruck, Anichstraße 35, 6020 Innsbruck, Tyrol, Austria; anna.joebstl@gmx.net (A.J.); piera.tierno@student.i-med.ac.at (P.M.T.); anna-katharina.gerstner@i-med.ac.at (A.-K.G.); gudrun.feuchtner@i-med.ac.at (G.M.F.); 2 Department of Internal Medicine I, Gastroenterology, Hepatology and Endocrinology, Medical University of Innsbruck, Anichstraße 35, 6020 Innsbruck, Tyrol, Austria; benedikt.schaefer@i-med.ac.at (B.S.); herbert.tilg@i-med.ac.at (H.T.)

**Keywords:** hepatocellular carcinoma, LI-RADS, computed tomography, segmentation, texture analysis

## Abstract

Background/Objectives: Hepatocellular carcinoma (HCC) is the most common primary malignant tumour of the liver. In a cirrhotic liver, each nodule larger than 10 mm demands further work-up using CT or MRI. The Liver Imaging Reporting and Data System (LI-RADS) is still based on visual assessment and measurements. The purpose of this study was to evaluate whether semi-automated quantification of visual LR-5 lesions is appropriate and can objectify HCC classification for personalized radiomic research. Methods: A total of 52 HCC patients (median age 67 years, 17% females, 83% males) from a retrospective data collection were evaluated visually and compared by the results using an oncology software with features of LI-RADS-based structured tumour evaluation and documentation, semi-automated tumour segmentation, and texture analysis. Results: Software-based evaluation of non-rim arterial-phase hyperenhancement (APHE) and non-peripheral washout, as well as the LI-RADS-score, showed no statistically significant differences compared with visual assessment (*p* = 0.2, 0.7, 0.17), with a consensus between a human reader and the software approach in 98% (APHE), 89% (washout), and 93% (threshold growth) of cases, respectively. The software provided automated LI-RADS classification, structured reporting, and quantitative features for HCC registries and radiomic research. Conclusions: The presented work may serve as an outlook for LI-RADS-based automated qualitative and quantitative evaluation. Future research may show if texture analysis can be used to foster personalized medical approaches in HCC.

## 1. Introduction

Hepatocellular carcinoma (HCC) is the most common primary malignant tumour of the liver, with an increasing incidence worldwide [1]. It is the fourth most common cancer and the second leading cause of cancer-related death, particularly in men [1]. Common causes for the development of HCC include chronic type B and type C hepatitis (HBV, HCV, each 40%), alcoholic liver disease (ALD, 11%), and metabolic-dysfunction-associated steatotic liver disease (MASLD), with the incidence of MASLD increasing, particularly due to obesity and diabetes, as well as immune system disorders [1]. The most important risk factor for HCC is cirrhosis, which occurs in 90% of cases [2], but the development of HCC in non-cirrhotic livers is increasing, especially in association with MASLD [3].

Imaging surveillance should be performed in patients with cirrhosis (Child–Pugh A and B, Child–Pugh C in liver transplantation), as well as in patients without cirrhosis but with stage F3 fibrosis or at particular risk for HCC [4]. The European Association for the Study of the Liver (EASL) recommends biannual liver ultrasound and optionally the use of magnetic resonance imaging (MRI) or computed tomography (CT) in patients awaiting liver transplantation or whose ultrasound quality is inadequate [4].

Any liver nodule larger than 10 mm requires further evaluation by CT or MRI [5]. CT with a late hepatic artery, portal venous, and delayed phase is the standardized imaging protocol [2,6], and MRI is usually reserved for difficult cases [6] or as an adjunct. LI-RADS (Liver Imaging Reporting and Data System)—first introduced in 2011 and most recently updated in 2018—was developed to standardize the reporting and data collection of CT and MR imaging in HCC. It is based on five main qualitative and quantitative criteria: size, non-rim hyperenhancement in the arterial phase (APHE), non-peripheral washout in the portal venous and/or delayed phase, enhancing capsule, and threshold growth [7]. APHE is assessed in the arterial phase and is defined as arterial contrast enhancement of the observed liver lesion that is superior to that of the background liver. It may be homogeneous or heterogeneous but must not take place primarily peripherally [8]. The lesion size, i.e., the largest diameter (including the capsule, if present), should be measured in the phase and at the level where the margins can be best delineated [8]. If possible, the arterial phase should not be chosen for size measurement due to potential overestimation. Non-peripheral washout is assessed in the portal venous and/or delayed phase and is defined as hypoenhancement of the observed liver lesion compared to the background liver and compared to an earlier phase. Therefore, at least two contrast phases are required for a comprehensive assessment. It may also be homogeneous or heterogeneous but again must not be observed primarily peripherally [8]. An enhancing capsule surrounds the observed liver lesion at least partially and is most strongly enhanced in the portal venous or delayed phase [8]. Threshold growth is defined as an increase in size of at least 50% in ≤6 months. Measurements must be performed in the same phase and in the same plane in both examinations [8]. In addition, there are more minor features favouring malignancy (e.g., subthreshold growth, non-enhancing capsule, and blood products in mass) and benignity (e.g., size stability > 2 years, size reduction) that allow an upgrading or downgrading of the LI-RADS category (LR) by one [8].

A lesion classified as LR-5 allows diagnosis and treatment as HCC without pathological confirmation. It requires APHE and a size of at least 10 mm, with washout, threshold growth, or an enhancing capsule as additional requirements. Currently, radiologists analyse imaging findings subjectively, including manual size measurements and visual comparisons of CT densities between liver parenchyma and liver lesions, to assess APHE and non-peripheral washout for LI-RADS classification. Software-based automated or semi-automated lesion segmentation could improve LI-RADS HCC categorization by quantifying tumour size and volume, analysing density and histogram texture, quantifying the washout and attenuation ratio, and calculating threshold growth. It could also reduce variability between findings. Furthermore, computer-assisted measurements can realize the full potential of LI-RADS based on structured documentation, data storage, and radiomics analysis for HCC registries. Structured reporting is now widely known and recommended because it improves the quality and completeness of radiology reports, as well as referring physician satisfaction and communication [9]. Structured reports must be consistently organized and aligned with the clinical question [9]. Due to their clear structure and predefined terminology, existing reporting systems, such as the various RADS systems, are particularly well-suited for the application of structured reporting [9]. In addition to routine clinical applications, other research projects or data registries can also benefit from structured reporting, as it enables data mining [9].

The aim of this study was to evaluate whether semi-automated quantification of visual LR-5 lesions is appropriate and can objectify HCC classification for personalized radiomic research.

## 2. Materials and Methods

This retrospective investigation represents a post hoc sub-analysis of a larger study cohort. The study was conducted in accordance with the Declaration of Helsinki and approved by the Institutional Review Board/Ethics Committee of Medical University of Innsbruck (EK Nr. 1344/2021, 23 February 2022). Due to the retrospective nature of the study, patient consent was waived by the Institutional Review Board/Ethics Committee of Medical University of Innsbruck.

Fifty-two HCC-patients from a retrospective data collection were included. Inclusion criteria were availability of a full CT-HCC protocol (consisting of a non-enhanced, an arterial, a portal venous, and a late venous phase) and at least one visually assessed LR-5 lesion. Additionally, we excluded six cases in which the semi-automated tumour segmentation did not work sufficiently. In cases of multifocal disease, we only included one visual LR-5 lesion per patient.

**Figure 1:**
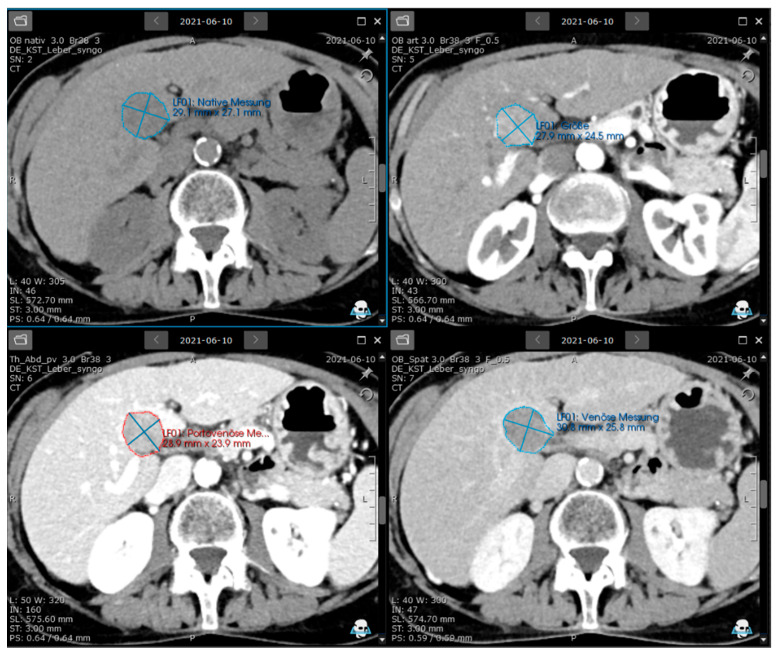
Screenshot from the software-assisted, semiautomatic segmentation of a LI-RADS-5 liver lesion in four different contrast phases. Upper left—native (29.1 mm × 27.1 mm); Upper right—arterial (27.9 mm × 24.5 mm); Lower left—portal venous (28.9 mm × 23.9 mm); and Lower right—late venous phase (30.8 mm × 25.8 mm).

Visual assessment of the LI-RADS features and score was taken over from the written reports, as each study had been carried out in the clinical routine. In a second step, an oncology software with features of LI-RADS-based structured tumour evaluation and documentation, semi-automated tumour segmentation, and texture analysis (mint Lesion™, Version 3.10.0—mint Medical GmbH, Heidelberg, Germany) was applied. Its 3D volume tool was applied for semiautomatic segmentation of the manually chosen lesions. This segmentation is suitable for homogeneous, well-defined lesions. The centre of the object was marked with the computer mouse. The mouse was then moved to draw a sphere around it, which initiated the segmentation process. The quality of the segmentation was verified visually in all image slices. If inaccurate, the segmentation could be manually adjusted by pressing or dragging along the edge. In approximately 50% of cases, manual corrections to the semi-automatic segmentation were necessary. However, these were minor and straightforward adjustments that required only a small amount of additional time. Measurements were taken in all CT phases (Figure 1). Additionally, for each contrast phase, the mean density of the background liver was determined as the average of one ROI (region of interest, 2 cm diameter) in the left and right liver lobes. The size and mean density of the lesion were automatically determined based on semi-automatic segmentation. If a prior examination was available, the presence or absence of threshold growth was automatically assessed. The other main and minor features (LI-RADS v2018) had to be entered manually into mint Lesion™ as “yes,” “no,” or “not evaluable.” Based on the semi-automatically determined size and threshold growth, as well as the manually entered main and minor features, mint Lesion™ automatically classified the lesion using LI-RADS v2018.

For this paper, we aimed to expand the computer-aided evaluation of liver lesions by additionally using Microsoft Excel, where we built a file that automatically calculated APHE and washout based on the exported csv (comma-separated-values format) CT density values (HU—Hounsfield Units), which had been measured in mint Lesion™ before. APHE was calculated as the difference between the mean density of the observed nodule and the background liver in the arterial phase (${HU}_{lesion,art}-{HU}_{liver,art}$). If the result was positive (hence, HU of the nodule was higher than HU of the background liver), APHE was given. Non-peripheral washout was calculated as the percentage attenuation ratio (PAR), as described by Liu et al. 2013, being the ratio between mean density of the background liver and of the lesion in the portal venous or late venous phase ($\frac{{HU}_{liver,mean,pv}}{{HU}_{lesion,mean,pv}}\;and\;\frac{{HU}_{liver,mean,del}}{{HU}_{lesion,mean,del}})$ [10]. For a result > 1, washout was accepted for the respective phase. Since the washout only needs to be present in one of the venous contrast phases, we used the higher of the two PAR values to demonstrate the washout.

Our file provided an automated LI-RADS classification based on the calculated APHE and washout values, as well as all other important and additional features that were saved in and exported from the oncology software. Regarding lesion size, we offered the option of selecting the respective contrast phase in which the lesion was best visible. A text module was created that could be easily used for structured reporting by copying and pasting.

### Statistical Analysis

Descriptive statistics were used to describe the basic data. To compare the results of visual and arithmetic assessments, we used the McNemar–Bowker Test and to compare the size between different contrast phases the sign test. Microsoft Excel was used for all the calculations and testing. A *p*-value of <0.05 was defined as statistically significant.

## 3. Results

### 3.1. Study Population

Of the 52 originally visually classified LR-5 lesions in 52 patients, six had to be excluded because the semi-automatic segmentation of the liver lesion failed. The reasons were limited visibility or blurred contours in inhomogeneous and irregular contrast-enhancing lesions. The software could not correctly identify the lesion edge. In such a case, the interpolating layer-by-layer 3D segmentation tool would have been required. However, we wanted to use the semi-automatic tool because it is most suitable for use in routine clinical practice. Ultimately, 46 cases were analysed. Eighty-three percent (38) were male, and seventeen percent (8) were female. The median age was 67 years, with a minimum of 42 and a maximum of 82 years.

Liver cirrhosis was present in 43 cases (93%). In the remaining three cases, a previous HCC had already been treated with radiofrequency ablation (RFA), transarterial chemoembolization (TACE), or hemihepatectomy. The causes of cirrhosis were MASLD in 32%, ALD in 28%, HCV in 16%, and HBV in 5%. Other or unknown causes were present in 19%.

All liver segments, except segments 1 and 4b, were affected, with most tumours (65%) located in the functional right lobe (segments 5–8), versus 35% in the functional left lobe (segments 1–4).

### 3.2. Arterial-Phase-Hyperenhancement (APHE)

**Figure 2:**
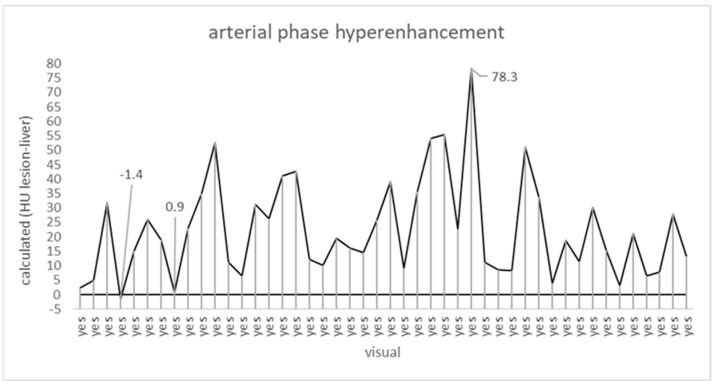
Comparison of visual (x-axis; “yes”/”no”) and arithmetic (y-axis; ∆ lesion vs. background liver) assessment of APHE. The horizontal line equals isoenhancement (∆ = 0). APHE—arterial-phase hyperenhancement.

As required for any LR-5 classification, visual assessment demonstrated APHE in all 46 cases. Arithmetic evaluation demonstrated APHE in 45 (98%) cases, but not in one. In this one case, the mean attenuation of the liver lesion was 1.4 HU lower than that of the background liver, while the mean attenuation of the other liver lesions was between 0.9 and 78.3 HU higher than that of the background liver (Figure 2). This deviation was not statistically significant (*p* = 0.2).

### 3.3. Non-Peripheral Washout

**Figure 3:**
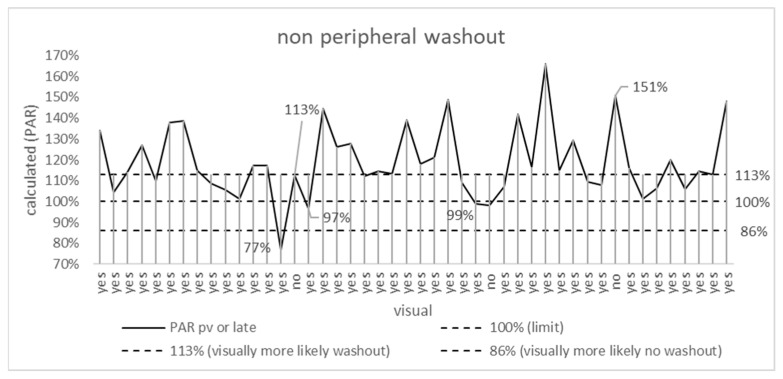
Highest PAR for portal venous and late venous phase. The dashed line at 100% marks the limit between washout (>100%) and no washout (≤100). The dashed lines at 86% and 113% mark areas in which visual assessment will more likely result in no washout and washout, respectively, as stated by Liu et al., 2013 [10]. PAR—percentage attenuation ratio. The x-axis shows the results of the visual assessment (washout “yes” or “no”).

**Figure 4:**
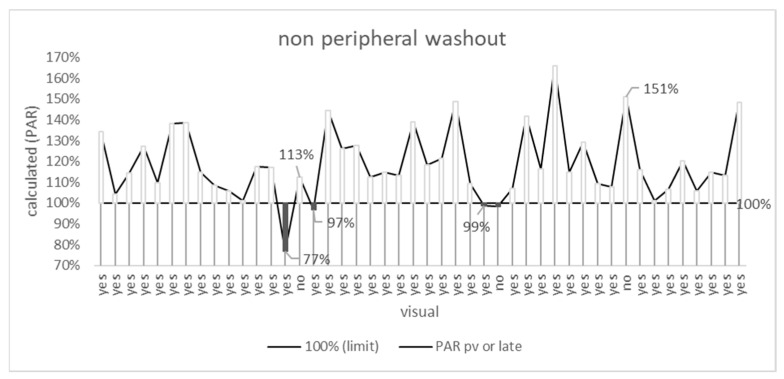
Difference between the calculated PAR and the 100% limit. For white bars, “PAR > 100%” (=washout) is true; for black bars, “PAR ≤ 100%”(=no washout) is true. PAR—percentage attenuation ratio. The x-axis shows the results of the visual assessment (washout “yes” or “no”).

In 87% (40), washout was seen visually and arithmetically, and in 2% (1), no washout was seen visually or arithmetically. In the remaining 11% (5), visual and arithmetic assessments differed (Figure 3 and Figure 4, Table 1).

Considering only the late venous phase, there was also accordance between visual and arithmetic results in 89%, regarding only the portal venous phase in only 74% (Table 1).

The difference between visual and arithmetic results was not statistically significant for portal venous (*p* = 0.18), late (*p* = 0.25), or the combination of both phases (*p* = 0.7).

For both visual and arithmetic assessment, non-peripheral washouts occur more frequently in the late venous phase (41 and 38, respectively) than in the portal venous phase (35 and 31, respectively). Washout was present only in the late venous phase in 8 and 11 cases, respectively, and only in the portal venous phase in 2 and 4 cases, respectively. A contrast-enhancing capsule was observed in nine cases (20%).

### 3.4. HCC Size

Lesion margins were best visible in the arterial phase in 50% of cases, in the late venous phase in 28%, in the portal venous phase in 20%, and in the native phase in 2%. In those best phases, 15 lesions (33%) had a maximum diameter between 10 and 19 mm, and 31 (67%) had a maximum diameter of 20 mm or more. None were smaller than 10 mm. In the arterial phase, 11 lesions (24%) had a maximum diameter between 10 and 19 mm, and 35 (76%) had a maximum diameter of 20 mm or more. None were smaller than 10 mm. This supports the statement that overestimation of size can occur in the arterial phase; however, in our small cohort, the difference was not significant (*p* = 0.13).

In 11 cases (24%), threshold growth was present on visual assessment, in 8 cases (17%), threshold growth was present on automated assessment, and all cases without prior examination in the last six months were rated as “no threshold growth”.

### 3.5. LI-RADS

Four different LI-RADS-classifications were calculated: visual and arithmetic assessment in the arterial phase (Table 2 and Table 3) and the best phase (Table 4 and Table 5).

The comparison of the visual and arithmetic assessment (Table 2 and Table 3) showed that four cases mathematically lost their LR-5 classification, in one case because mathematically there was no APHE, in two cases because mathematically there was no washout, and in one case because there was no threshold growth in the automatic assessment. Comparison between sizing in the arterial phase and the best phase showed no significant changes in the visual assessment group (Table 2 and Table 4), as four lesions were smaller in the best phase (10–19 mm instead of over 20 mm) but remained LR-5 due to their adjacent features.

However, comparing the size determination based on the arterial or best phase resulted in a practically relevant change in the computationally assessed cases (Table 3 and Table 5), as the one lesion without computational APHE was also smaller in the best phase (less than 20 mm instead of more than 20 mm in the arterial phase)—therefore, it was classified only as LR-3. Three other lesions were also smaller in the best phase, but due to the adjacent features, there was no change in classification (they remained at LR-5). Comparing the LI-RADS classification based on the best phase size for both visual and computational assessment (Table 4 and Table 5), there were no statistically significant differences (*p* = 0.17).

### 3.6. Quantitative Analysis and Structured Reporting

**Figure 5:**
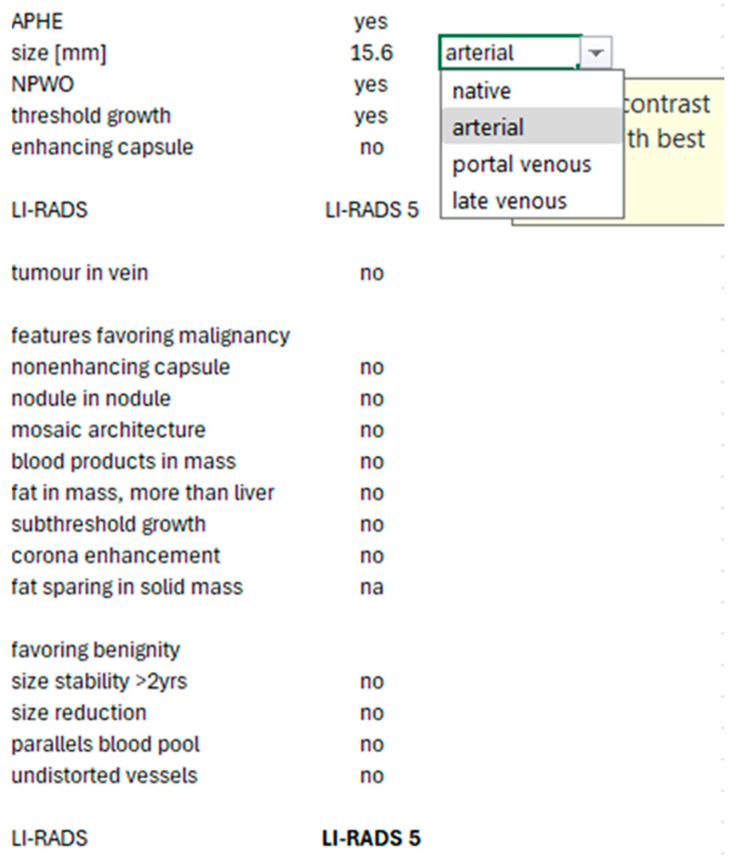
Example of a text module based on the size (best contrast phase can be chosen), density (liver and lesion), and major as well as ancillary features exported from the oncology software with automated calculation of APHE, non-peripheral washout, and LI-RADS using Microsoft Excel.

Since significant differences in LI-RADS categorization can occur depending on the selected contrast phase, we implemented a manual selection of the best phase for sizing in our Excel-based text module. We used the automatic calculations of APHE and non-peripheral washout described above. The major and minor features were automatically imported from the oncology software (see the example in Figure 5).

## 4. Discussion

We present data on a semi-automated quantification of visual LR-5 lesions aiming to objectify HCC classification for personalized radiomic research. In our cohort of 46 LR-5 lesions, margins were best seen on the arterial phase (50%), followed by the late venous phase (28%) and the portal venous phase (20%). Use of arterial or best phase for size measurement could lead to a relevant change, as one lesion without calculated APHE was smaller on the best phase (less than 20 mm instead of more than 20 mm on the arterial phase) and therefore classified as LR-3. Three further lesions were smaller in the best phase but because of the additional features remained LR-5. This highlights the importance of the arterial phase for characterization of HCC.

For both visual and arithmetic assessment, non-peripheral washout was more often observed in the late venous phase (41 and 38, respectively) than in the portal venous phase (35 and 31, respectively). It may be occasionally observed either on the late venous phase or the portal venous phase instead of both phases. An enhancing capsule as an important ancillary feature was seen in 20%.

Our study demonstrated consensus between a human reader and the semi-automated quantification in 98% for APHE, 89% for washout, and 93% for threshold growth. By comparison, Ehman et al. measured human interobserver agreement regarding the major features of the LI-RADS-classification. For CT examinations, they showed consensus regarding the presence or absence of APHE in 96%, non-peripheral washout in 90% and an enhancing capsule in 82% [11].

Using the semi-automated classification in our study, four cases lost their LR-5 status: one due to a lack of APHE calculation, two due to a lack of washout, and one due to a lack of calculated threshold growth rates. According to the management suggestions by AASLD (American Association for the Study of Liver Diseases) and LI-RADS in consensus, LR-5 refers to confirmed HCC and requires multidisciplinary discussion for consensual treatment. LR-4 requires multidisciplinary discussion for a tailored investigation, which may include a biopsy. Thanks to this algorithm with the multidisciplinary discussion of LR-4 and LR-5 lesions, the clinical impact of the small number of discordant cases remains negligible and may not alter the clinical outcome.

Automated data export to a LI-RADS reporting tool in Microsoft Excel, which can calculate APHE, washout, and the respective LI-RADS score, resulted in a text module containing all the information needed for a structured LI-RADS report. Especially with complex classification systems like LI-RADS, the use of software support offers advantages, as it offers a simplified approach, as the radiologist only needs to click through the various features without having to memorize them all, and as the final interpretation and classification of the result is automated. All data can be saved and used for reading and structured reporting, as well as for research and cancer registry development. The qualitative and quantitative evaluation offers great potential for personalized diagnosis, treatment, and post-treatment disease prognosis. Integrating structured reporting based on a software-assisted LI-RADS report generator into clinical workflows can increase efficiency and save time and effort. Furthermore, it can improve the standardization of radiological reports and enhance communication with referring physicians and patients.

On the other hand, using additional computer programs in addition to regular diagnostic image evaluation can be labour-intensive and often requires additional training. Users must be aware of the strengths and weaknesses of software tools. Semi-automatic segmentation and measurements can be prone to errors in lesions with blurred contours or low contrast with the surrounding tissue—we excluded 11% of our patients due to inadequate semi-automatic segmentation. Manual segmentation is labour-intensive and susceptible to inaccuracies. Furthermore, every manual action negates the benefit of higher interobserver agreement that could result from automated measurements.

### Study Limitations

A limitation of this paper is that it only includes 46 evaluable LR-5 lesions, which limits statistical power and generalizability. Inclusion of only category 5 lesions precludes assessment of false positives or differentiation among lower LI-RADS categories. However, the aim was to evaluate whether semi-automated quantification of visual LR-5 lesions is appropriate and can objectify HCC classification. Future studies should extend the cohort to include a wider spectrum of LI-RADS categories to better evaluate diagnostic performance and false positive rates.

The LR-5 lesions were exported from retrospective image reports. However, this introduces potential study bias, as even when adhering to the strict rules of the LI-RADS diagnostic table, one radiologist may have different experience than another, which could therefore lead to different reporting results. Blind review of the studies with at least two different radiologists would be required to calculate the agreement of the findings and compare the results with the software results.

As described by Liu et al., we used the average CT densities of liver parenchyma and liver lesions for our calculations [10]. However, according to the LI-RADS definitions, homogeneous density changes are not required for APHE and non-peripheral washout [8]. Non-peripheral and focal hyperenhancement or washout processes may be better depicted by values other than the average. On the other hand, average density measurements alone may not allow assessment of a contrast-enhancing capsule or focal and heterogeneous enhancement.

Future research may demonstrate whether the variability between human and computer-assisted readers can be further reduced by using other statistical approaches or even radiomics values to distinguish, for example, between generalized or focal and central or peripheral density changes. Such research could also increase the detection rate and diagnostic accuracy. It could offer important prospects for AI-based automated LI-RADS-based qualitative and quantitative assessment of HCC. Clinically relevant HCC subtypes, depending on volume, shape and enhancement patterns, texture signatures, and dynamic changes during post-treatment monitoring and follow-up, could enable personalized medical approaches for HCC.

## Figures and Tables

**Table 1:** Fourfold table showing agreement between visual and assessment regarding non-peripheral washout in portal venous, late venous, and both phases, respectively.

Portal Venous Phase (*p* = 0.18) Late Venous Phase (*p* = 0.25) Both Phases (*p* = 0.7)		Arithmetic	
		Washout	No Washout
visual	washout	portal venous 59%	portal venous 17%
		late venous 80%	late venous 9%
		both phases 87%	both phases 7%
	no washout	portal venous 9%	portal venous 15%
		late venous 2%	late venous 9%
		both phases 4%	both phases 2%

**Table 2:** Distribution of visually assessed LI-RADS scores (LR) in the arterial phase. Yellow—LR-3; orange—LR-4; red—LR-5. LI-RADS—Liver Imaging Reporting and Data System.

Arterial Phase Hyperenhancement (APHE)		No APHE		Nonrim APHE			
		0		46			
Observation size (mm)		<20 mm	≥20 mm	<10 mm	10–19 mm		≥20 mm
		0	0	0	11		35
Count additional features: Enhancing “capsule”; Nonperipheral “washout”; Threshold growth	None	0	0	0	0		0
	One	0	0	0	0	6	35
	≥Two	0	0	0	5		

**Table 3:** Distribution of arithmetically assessed LI-RADS scores (LR) in the arterial phase. Yellow—LR-3; orange—LR-4; red—LR-5. LI-RADS—Liver Imaging Reporting and Data System.

Arterial Phase Hyperenhancement (APHE)		No APHE		Nonrim APHE			
		1		45			
Observation size (mm)		<20 mm	≥20 mm	<10 mm	10–19 mm		≥20 mm
		0	0	0	11		34
Count additional features: Enhancing “capsule”; Nonperipheral “washout”; Threshold growth	None	0	0	0	0		2
	One	0	1	0	1	6	32
	≥Two	0	0	0	4		

**Table 4:** Distribution of visually assessed LI-RADS scores (LR) in the best phase. Yellow—LR-3; orange—LR-4; red—LR-5. LI-RADS—Liver Imaging Reporting and Data System.

Arterial Phase Hyperenhancement (APHE)		No APHE		Nonrim APHE			
		0		46			
Observation size (mm)		<20 mm	≥20 mm	<10 mm	10–19 mm		≥20 mm
		0	0	0	15		31
Count additional features: Enhancing “capsule”; Nonperipheral “washout”; Threshold growth	None	0	0	0	0		0
	One	0	0	0	0	9	31
	≥Two	0	0	0	6		

**Table 5:** Distribution of arithmetically assessed LI-RADS scores (LR) in the best phase. Yellow—LR-3; orange—LR-4; red—LR-5. LI-RADS—Liver Imaging Reporting and Data System.

Arterial Phase Hyperenhancement (APHE)		No APHE		Nonrim APHE			
		1		45			
Observation size (mm)		<20 mm	≥20 mm	<10 mm	10–19 mm		≥20 mm
		0	0	0	14		31
Count additional features: Enhancing “capsule”; Nonperipheral “washout”; Threshold growth	None	0	0	0	0		2
	One	1	0	0	1	9	29
	≥Two	0	0	0	4		

## Data Availability

The data presented in this study are available on request from the corresponding author due to legal reasons.

## Abbreviations

The following abbreviations are used in this manuscript:

ALD	Alcohol-associated liver disease
APHE	Arterial-phase-hyperenhancement
AASLD	American Association for the Study of Liver Diseases
CT	Computed tomography
EASL	European Association for the Study of the Liver
HCC	Hepatocellular carcinoma
HU	Hounsfield Units
LI-RADS	Liver Imaging Reporting and Data System
MASLD	Metabolic-dysfunction-associated steatotic liver disease
MRA	Magnetic resonance imaging
PAR	Percentage attenuation ratio
ROI	Region of interest
VOI	Volume of interest
